# Measurements of total and integrated HIV DNA demonstrate sporadic blips of unintegrated HIV DNA in HIV-positive patients on HAART

**DOI:** 10.1186/1758-2652-13-S3-O16

**Published:** 2010-11-04

**Authors:** A Mexas, E Graf, L Agosto, JJ Yu, M Pace, M Liszewski, S Migueles, M Connors, U O’Doherty

**Affiliations:** 1 Uninversity of Pennsylvania, Pathology and Laboratory Medicine, Philadelphia, USA; 2 NIAID, NIH, Bethesda, USA

## Background

How HIV reservoirs are maintained on HAART remains unclear. Ongoing replication may contribute to reservoir maintenance and may occur at a low level that is not easy to detect. In the absence of ongoing replication, unintegrated HIV DNA should be lost over time; thus, monitoring total and integrated DNA may address this question.

## Methods

We developed and validated a sensitive and precise assay to measure HIV integration. Total and integrated HIV DNA were then measured in a cross-sectional series of patients on HAART and in two patients longitudinally. The ratios of total over integrated were then calculated to determine the extent of unintegrated HIV DNA present in patient samples. The errors in the ratios were calculated to determine the lowest level of excess unintegrated HIV DNA that we could confidently detect.

## Results

All HIV-positive patients off HAART have an excess of unintegrated HIV DNA. Surprisingly, we found a statistically significant excess of unintegrated HIV DNA in three of seven well-suppressed patients in the cross-sectional study. We detected an excess of unintegrated HIV DNA sporadically in both of the patients that we evaluated longitudinally, with two of eight positive time points in one patient and four of 10 in the other. Notably, for most time points in the longitudinal analysis, the levels of total and integrated HIV DNA were not significantly different.

## Conclusions

Our data suggest that unintegrated HIV DNA can be detected sporadically in patients on HAART. Further work is required to determine the meaning of an excess of unintegrated HIV DNA. We speculate that an excess of unintegrated HIV DNA may correlate with a burst of ongoing replication. If so, these methods could aid in the evaluation of therapies that aim to intensify HAART or flush out viral reservoirs in patients on HAART.

